# Genomic and virulent characterization of a duck-associated *Salmonella* serovar Potsdam from China

**DOI:** 10.1016/j.psj.2024.104646

**Published:** 2024-12-05

**Authors:** Hongli An, Xiamei Kang, Chenhu Huang, Chenghao Jia, Jiaqi Chen, Yingying Huang, Qianzhe Cao, Yan Li, Biao Tang, Min Yue

**Affiliations:** a Department of Veterinary Medicine, Zhejiang University College of Animal Sciences, Hangzhou 310058, China; b Hainan Institute of Zhejiang University, Sanya 572025, China; c State Key Laboratory for Diagnosis and Treatment of Infectious Diseases, National Clinical Research Center for Infectious Diseases, National Medical Center for Infectious Diseases, The First Affiliated Hospital, Zhejiang University School of Medicine, Hangzhou 31003, China; d Key Laboratory of Systems Health Science of Zhejiang Province, School of Life Science, Hangzhou Institute for Advanced Study, University of Chinese Academy of Sciences, Hangzhou 310024, China

**Keywords:** *Salmonella*, Duck, Virulence, Potsdam, Serovar, Host adaptation

## Abstract

*Salmonella*, a common zoonotic pathogen, is a significant concern for public health, particularly when it contaminates animal-borne products. The potential for *Salmonella* to infect duck embryos and disrupt their normal development not only causes substantial economic losses for the industry but also poses a severe threat to public health. However, there is a lack of understanding about the prevalence of *Salmonella* in duck embryos and their potential public health implications. Our study aims to fill this gap by providing genomic features of the antimicrobial resistance and virulence potential of *Salmonella* isolates from dead duck embryos using whole-genome sequencing and *in silico* toolkits. We also sought to assess the virulent characterization of the major serovar isolates by experimental infection of chicken and duck embryos. Our investigation of 195 duck embryo eggs led to the isolation of 40 (20.51%) *Salmonella* strains, with *Salmonella* serovar Potsdam being the most prevalent serovar. Most isolates were resistant to streptomycin (57.3%) and nalidixic acid (50%). Notably, our findings demonstrated that *S*. Potsdam exhibited a preference for ducks over chickens, suggesting potential host specificity. Additionally, global phylogenomic analysis, incorporating 180 global genomes, revealed a predominant association of *S*. Potsdam with ducks, supporting an adaptive process specific to the waterfowl. This study determined *Salmonella* serovars and antimicrobial resistance profiles in dead duck embryos, revealing a rare *Salmonella* serovar Potsdam with a potential for duck adaption.

## Introduction

The consumption of duck meat has increased in recent years, with consumers demonstrating a growing acceptance of it due to its organoleptic properties, high concentration of unsaturated fatty acids, and other nutritional attributes (Zhu et al., 2022). However, *Salmonella* infections in ducks can result in significant economic losses, threaten food safety, and represent a primary concern for the meat duck farming industry (Garvey et al., 2013; Paudyal et al., 2018). Infections caused by *Salmonella* are common in waterfowl, especially in intensive farming (Li et al., 2022a; Tang et al., 2023; Wang et al., 2019; Xu et al., 2020b, 2021; Zhou et al., 2022). Despite the fact that duck meat is widely consumed and China has become the world's largest producer of farmed waterfowl, there are still significant research gaps in the knowledge of *Salmonella* serovars detected in waterfowl, which require further investigation.

The infection of duck embryo eggs with *Salmonella* can occur via two distinct transmission pathways: horizontal and vertical (Elshebrawy et al., 2021). Contaminated eggs may enter the hatchery, resulting in vertical transmission, if the breeder farm supplying the hatchery is itself contaminated (Cardoso et al., 2021). Alternatively, horizontal transmission may occur when the eggshells are externally contaminated, potentially due to contact with the farm environment (Song et al., 2022), highlighting the need for strict hygiene practices during the farming processes. During the collection of eggs or within the hatchery environment, the *Salmonella* present on the eggshells can be distributed throughout the environment. *Salmonella* may penetrate the eggshell, particularly if the shell is of poor quality or ruptured. In the case of unformed eggs within the reproductive tract, the bacteria can colonize the yolk, white or shell membranes, resulting in direct contamination of the duck egg (Gast et al., 2024).

The primary focus of *Salmonella* contamination is the breeding flocks, feed mills and hatcheries. *Salmonella* typically enters the hatchery through contaminated embryo eggs and can become a resident contaminant in the ventilation system (Kang et al., 2024; Pijnacker et al., 2019). In some cases, it may become a resident contaminant in ventilation systems where cleaning and disinfection are less easily carried out. Some *Salmonella* serovars (e.g., *Salmonella* Senftenberg) have been observed to survive longer in hatcheries than others, which may be due to their ability to form biofilms (Kalily et al., 2016); this can cause severe economic losses if hatcheries become contaminated. Consequently, the precise identification of antimicrobial resistance (AMR) and the environmental adaptation of *Salmonella* strains isolated from duck embryos furnishes pivotal information that can serve as the foundation for the targeted treatment of dead duck embryos and the implementation of preventive measures.

This study collected duck embryo eggs from a duck hatchery, the aim of the study was to determine the prevalence of *Salmonella* in the duck embryo eggs and to characterize *Salmonella* isolates. We also intended to understand the virulence of the major serovar of *Salmonella* Potsdam and the global information, which would also be of interest and value in understanding the spread of *Salmonella* Potsdam and in the field of human public health. Furthermore, our research involved the examination of antibiotic resistance and environmental tolerance. The primary serovar of *Salmonella* Potsdam was subjected to virulence assays in chicken and duck embryo models. Moreover, the complete genome of the *S*. Potsdam was sequenced, which facilitated the elucidation of the diverse host virulence properties.

## Results

### *Salmonella* from duck embryos

**Fig. 1:**
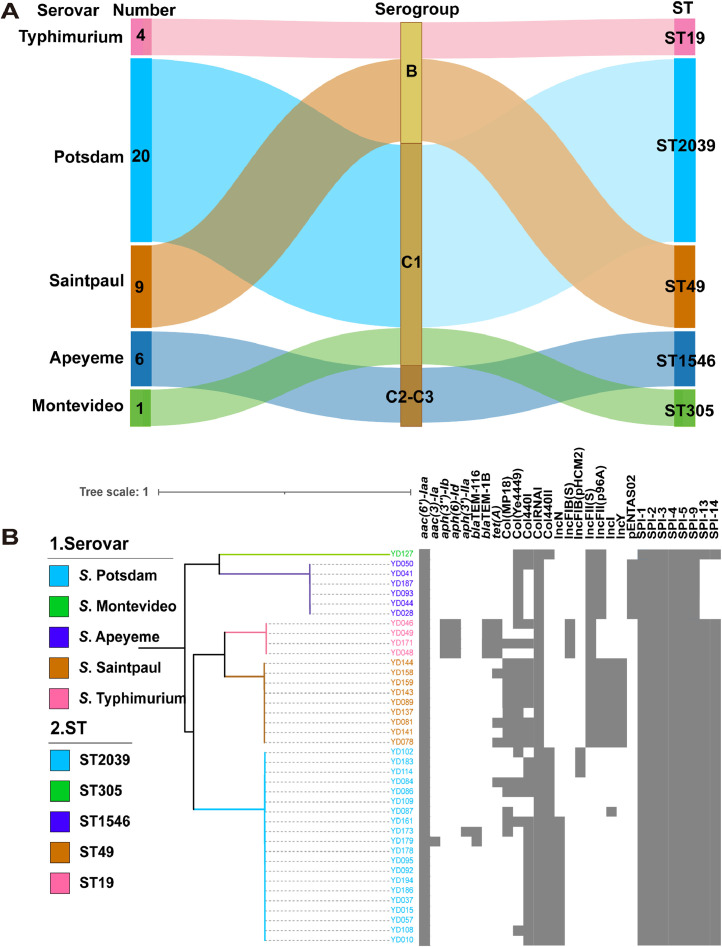
**The *S*. Potsdam is a dominant serovar isolated from duck embryo eggs.** (**A**) The serovars, serogroups and multilocus sequence typing (MLST) of *Salmonella* isolates, sequence type (ST) 2039 is the most predominant (n = 20). (**B**) The maximum‐likelihood phylogenetic tree among the *Salmonella* isolates, the grey colour indicates the antimicrobial resistance gene, plasmid replicon type and *Salmonella* pathogenicity islands (SPIs).

In this study, a total of 40 (20.51%) *Salmonella* isolates were isolated from 195 samples collected from the Zhejiang province of China, belonging to five serovars. Among them, the serovars *S*. Potsdam exhibited the highest isolation rate (n = 20, 50%), followed by *S*. Saintpaul (n = 9), *S*. Apeyeme (n = 6), *S*. Typhimurium (n = 4), *S*. Montevideo (n = 1) (Fig. 1**A**). All of them were grouped into three different serogroups: O:4 (B) (13, 32.5%), O:8 (C2-C3) (21, 52.5%), O:7 (C1) (6, 15%). Multilocus Sequence Typing (MLST) analysis revealed that sequence type (ST) 2039 predominated (n=20), exclusively associated with the *S*. Potsdam serovars, followed by ST49 (n = 9), ST1546 (n=6), ST19 (n=4) and ST305 (n=1). Generally, there are strong correlations among serovars, serogroups and sequence types. To investigate the genetic relationship of these isolates, phylogenomic analyses were conducted using Whole-Genomic Sequencing (WGS) data for isolates (Fig. 1**B**). Prediction of plasmid types of the studied *Salmonella* isolates was performed using the Plasmidfinder database. The most prevalent plasmid incompatibility group was *ColRNAI* (n=40), *Col440I* (n=30), and *Col(MP18)* (n=23), followed by *Col440II, Col(Ye4449), IncFIB(S), IncFIB(pHCM2), IncFII(S), IncFII(p96A), IncI, IncN, IncY,* and *pENTAS02*. In addition, *S*. Apeyeme had a greater diversity of plasmid types, whereas *S*. Potsdam carried very few plasmids. These serovars have similar *Salmonella* Pathogenicity Island (SPI), but *S*. Apeyeme does not have SPI-13 and SPI-14 (Fig. 1**B**).

### Antimicrobial-resistant susceptibility and environmental tolerance

**Fig. 2:**
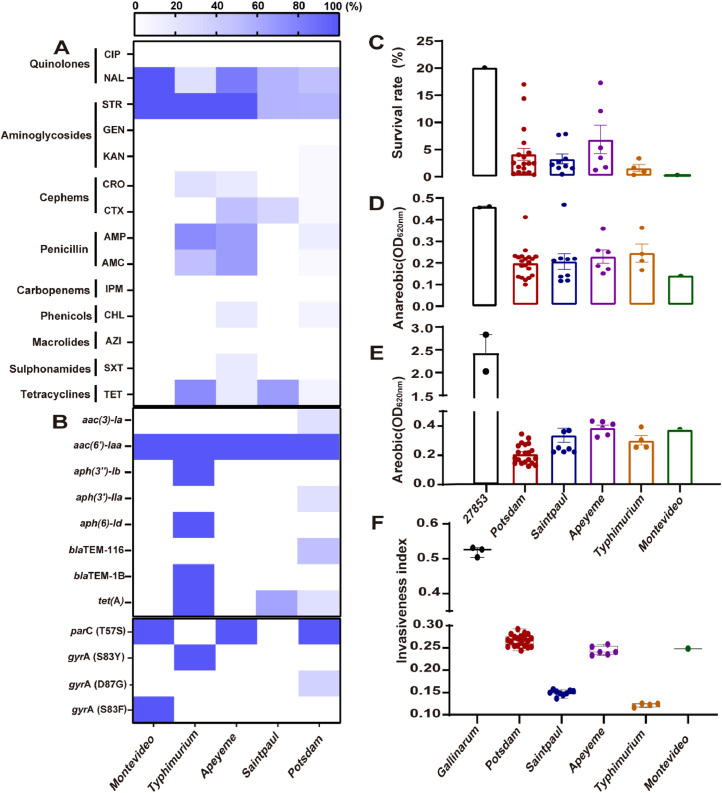
**Antimicrobial resistance phenotypes and genotypes, environment adaptation capacity, and invasion index of 40 *Salmonella* isolates.** (**A**) The antimicrobial susceptibility and resistance determinants of the *Salmonella* isolates. The colour indicates the rate of antimicrobial resistance to nine types of antibiotics. (**B**) The resistance determinants of the *Salmonella* isolates. Antimicrobial resistance genes (ARG) and associated gene mutations were identified with ResFinder (ver. 3.2). (C) The survival rate of drying, the isolated bacterial culture was adjusted to OD620 in 0.1 and incubated in relative humidity of 36%, the survivals were calculated. (D-E) The biofilm-forming capacity, the biofilms of 40 isolates are tested by static growth in Trypticase Soy Broth (TSB) at 37 °C for 48 h under aerobic and anaerobic conditions. (F) Invasive index of 40 *Salmonella* isolates, the invasion index uses DeltaBS and random forests to discern gene degradation patterns linked to invasiveness in *Salmonella*, thereby suggesting niche-specific or host-specific adaptation. A *Salmonella* Gallinarum strain R51 was used as a control. Dots indicate the invasive index of each isolate.

This study used the minimum inhibitory concentration method to test the antimicrobial resistance susceptibility of 40 *Salmonella* isolates to 14 antimicrobial agents. Our results showed that the majority of the isolates were highly resistant to aminoglycosides like streptomycin (STR) and quinolones like nalidixic acid (NAL) (Fig. 2**A**). In addition, *S*. Typhimurium exhibited high resistance to antimicrobial agents, such as ampicillin (AMP) and tetracycline (TET), while *S*. Potsdam showed a low resistance rate.

To understand the relationships between resistance phenotype and the genetic determinants, the antimicrobial resistance (AMR) patterns of these 40 isolates based on the WGS data were further analyzed. Eight antimicrobial resistance genes (ARG) were identified (Fig. 2**B**), which can be categorized into three classes, including *aac(3′)-Ia, aac(4′)-IIa, aph(3′')-Ib, aph(3′)-IIa, aph(6′')-Id* for aminoglycosides, *bla*_TEM-116,_ *bla*_TEM-1B_ for β-lactams and *tet*(A) for tetracyclines. *S*. Typhimurium and *S*. Potsdam possess five different ARGs, while the other three serovars carried only one or two ARGs (Fig. 2**B**). Moreover, we also detected the prevalence of genetic mutations that are linked with antimicrobial resistance. The *gyrA* gene mutations were identified in *S*. Typhimurium, *S*. Potsdam and *S*. Montevideo, while the *parC* gene was present in *S*. Typhimurium, *S*. Potsdam and *S*. Apeyeme (Fig. 2**C**).

Furthermore, we investigated the environmental tolerant capabilities of the 40 isolates under environmental stress conditions. After exposure to dry conditions, *S*. Apeyeme exhibited strong survival ability, followed by *S.* Potsdam (Fig. 2**D**), *S*. Potsdam exhibited the weakest biofilm formation ability under aerobic conditions (Fig. 2**E**). The invasion index based on the genomic dataset was also calculated. We found that the invasion indexes of *S.* Potsdam were higher than those of the other serovars (Fig. 2**F**), suggesting limited environmental adaptation capabilities for *S.* Potsdam. This might partially explain why *S.* Potsdam showed a low ability to form biofilms under aerobic conditions.

### Prediction of virulence genes

Our results show that 120 different virulence genes were detected, representing the different virulence and pathogenic mechanisms among *Salmonella* isolates (**Supplemental Table S2**). Fimbrial adherence determinants related genes, were detected in *S*. Typhimurium (100%); however, the *pef* genes (*pefA, pefB, pefC,* and *pefD*), encoding fimbriae, were lacking in other serovars, the *S*. Montevideo didn't detect the *lpf* genes (*lpfA, lpfB, lpfC, lpfD*). The secretion system-related genes (*spvC, spcR, seeI*), were detected in *S*. Typhimurium (100%), other serovars are rarely detected. The *rck* and *sodCI* gene, encoding serum resistance and stress adaptation, were detected in *S*. Typhimurium (100%), this serovar may be more likely to survive in the environment. *S*. Montevideo carried some genes with no detectable virulence in other serovars, e.g. *cdtB*, which codes for toxin, and *faeC, faeD*, but this serovar had the fewest virulence genes detected, which may be one of the reasons for the low isolation rate of this serotype. *S*. Potsdam carried fewer virulence factors, but it's the main isolate from dead duck eggs, it's worth thinking about its level of toxicity.

### *S*. Potsdam virulence in chickens and ducks

**Fig. 3:**
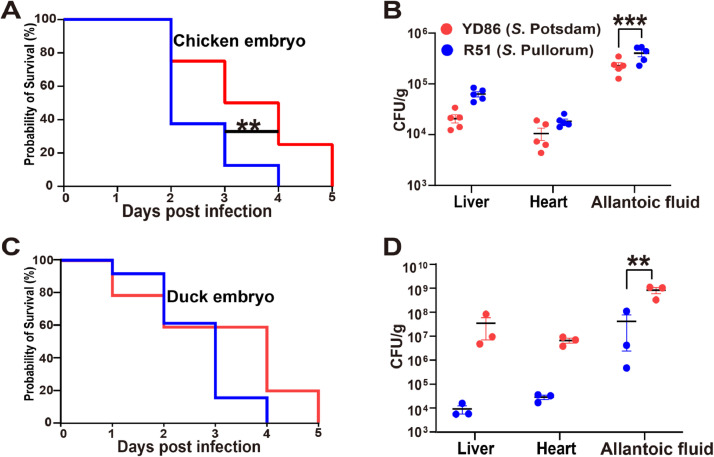
Virulence variability of Salmonella Potsdam in chicken and duck embryos. Increased virulence of Salmonella Potsdam to duck embryos and ducks. (**A**) The survival curves of chicken embryos infected with Potsdam isolates (n = 10), the highly specific *Salmonella* Pullorum named “R51” had a high virulence in chicken embryos. (**B**) The infected chicken embryo's liver, heart and allantoic fluid samples were harvested and tested for bacterial loads at 3 dpi (n = 5), the R51 had higher bacterial loads than YD86 in the allantoic fluid. (**C**) The survival curves of duck embryos infected with Potsdam isolates (n =10), *Salmonella* Potsdam named “YD86” had increased virulence in the duck embryos. (**D**) The infected duck embryo's liver, heart, and allantoic fluid samples were harvested and tested for bacterial loads at 3 dpi (n = 3), the YD86 had higher bacterial loads than R51 in the duck embryos. All the experiments were triplicated with biological replicates. Data are presented as mean ± SD. The *P*‐values for B and D were calculated using an unpaired t‐test (*P < 0.05). Dots indicate the bacterial loads of each isolate.

The dynamic virulence features of *S*. Potsdam (YD86) were evaluated using chicken and duck embryos, with *Salmonella* Pullorum strain (R51), which is highly specific to fowl, serving as a control (Zhou et al., 2023). YD86 demonstrated lower pathogenicity in the chicken embryo model than R51, the survival rate was markedly lower than R51 at five days post-infection (Fig. 3**A**), and the bacterial load in the liver and heart of infected chicken embryos was significantly lower than R51 at five days post-infection. The bacterial load of R51 was considerably higher than YD86 in the allantoic fluid of chick embryos. The allantoic fluid plays an essential role in embryo development, some nutrients secreted in the allantoic fluid, such as proteins and peptides, can be digested to provide free amino acids, which can be reabsorbed by the allantoic membrane and redirected to the embryo through the villous urothelial plexus. The amino acids can be reabsorbed by the allantoic membrane and redirected to the embryo through the chorionic allantoic capillary plexus, which results in R51 being more virulent in chicken embryos (Fig. 3**B**).

To ascertain whether the virulence of *S*. Potsdam is augmented in the waterfowl, the survival rates of YD86 and R51 were evaluated in the duck embryos. No significant discrepancy was observed in the survival rate of YD86 and R51 at five days post-infection (Fig. 3**C**). YD86 exhibited a pronounced capacity for colonization in duck embryos, with demonstrated significantly elevated bacterial loads in the liver and heart relative to R51 (Fig. 3**D**). This may indicate that *S*. Potsdam is more susceptible to infection in the waterfowl. In conclusion, the low level of virulence exhibited by YD86 in chicken embryos was reversed in duck embryos, which may be indicative of the host preferential virulence to waterfowl.

### Global genomics in *Salmonella* Potsdam

**Fig. 4:**
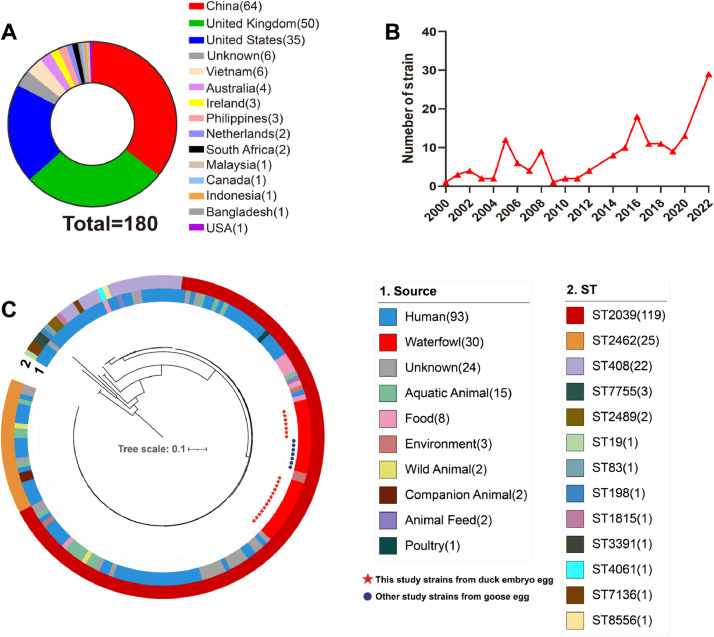
A maximum‐likelihood phylogenetic tree of 180 global S. Potsdam isolates. (**A**) 180 genome geographic location, with China being the country with the highest number of isolates of this strain. Using 180 global *Salmonella* Potsdam genomes, 20 of these strains were isolated for this study, while the remaining 160 were retrieved from the EnteroBase. (**B**) The temporal isolated strains trend among *S*. Potsdam population. (**C**) Source and MLST of the isolates were presented, isolated mainly from humans and waterfowl; the main popular ST type is ST2039. The time‐calibrated Bayesian phylogenetic tree was built based on the Core SNPs of *S*. Enteritidis P125109 strains by BEAST2 v2.6.3 (GTR substitution model, 4 gamma category count, relaxed clock log‐normal model, and Coalescent Bayesian Skyline tree prior model). Red star marks this study isolates, dark blue marks other study strains from goose egg. The number of examined isolates is marked in the bracket.

To investigate the genomic relationships between the studied isolates and the global strains, we analyzed the core single-nucleotide polymorphism (SNP). Using 180 global *Salmonella* Potsdam genomes (**Supplementary Table 1**), 20 of these strains were isolated for this study, while the remaining 160 were retrieved from the EnteroBase. *S*. Potsdam had the highest number of isolates from China, immediately followed by the United Kingdom (Fig. 4**A**). More *S*. Potsdam strains were isolated over time, especially after 2019 (Fig. 4**B**). Although the most frequent sources of strains are humans, followed by waterfowl (Fig. 4**C**), *Salmonella* infections in humans are mainly associated with direct consumption of contaminated waterfowl or other animals. There have been multiple reports provided prevalent evidence that linked this serovar with waterfowl and water (Guan et al., 2022; Su et al., 2011; Wang et al., 2020), which supports our conclusion, but it has not been uploaded to a public database. In this study, the strains we isolated were very closely associated with other strains isolated from goose eggs previously reported in China, possibly due to the epidemic spread of the strain (Guan et al., 2022). China is the preeminent duck-producing country in the world, with over 60% (712.4 million heads) of the global duck population reared in this country in 2019, this serovar may persist for extended periods in hatcheries causing economic losses. ST2039 was the popular type in recent years (Wang et al., 2020; Yang et al., 2020). In this study, all the strains we isolated were ST2039. The prevalence of ST2039 in duck embryos suggests a strong selective advantage for this serovar within the duck population.

## Discussion

*Salmonella* remains a significant zoonotic pathogen, which is often transmitted to humans through contaminated animal-borne products, including waterfowl products (Li et al., 2022b; Paudyal et al., 2020). Therefore, monitoring the prevalence of *Salmonella* in waterfowl is one of the essential components for public health, given the large number of foodborne diseases and associated outbreaks attributed to animal-borne products (Huang et al., 2024; Teng et al., 2024; Wang et al., 2024a). While previous studies have mainly identified *S*. Enteritidis and *S*. Typhimurium as the main serovars of *Salmonella* in ducks (Adzitey et al., 2012; Yang et al., 2019). Here, our finding reveals the emergence of *S.* Potsdam as the most prevalent serovar in duck embryo eggs, indicating a previously underestimated threat. The traditional culture method for identifying *Salmonella* serovar may yield false-negative results due to the growth differences among serovars and genotypes. WGS has emerged as a suitable, cost-effective method for epidemiological analysis of foodborne pathogens (Tang et al., 2023); routine WGS surveillance can significantly enhance our ability to monitor and recognize new threats along the food chain (Chen et al., 2024; Tang et al., 2023). Our findings underline the potential impact of this finding on public health and the urgency of addressing this issue.

In this study, the susceptibility of *Salmonella* isolates to 14 antibiotics belonging to 9 different classes was assessed. The results demonstrated that the isolates exhibited resistance to quinolones, aminoglycosides, and beta-lactams. Quinolones are commonly supplemented in poultry feeds in China, which may exert selective pressure on the pathogen and consequently lead to the development of quinolone resistance in *Salmonella* (Wang et al., 2024b; Xu et al., 2020a). *S*. Typhimurium had a higher rate of resistance gene and may be more easily transmitted in the environment. The ability of the pathogen to form biofilms is a characteristic that proves the virulence of a strain, also strains with a strong biofilm-forming ability are more difficult to remove from the surface of farm environments. Among our strains, *S*. Apeyeme resisted desiccation better and *S*. Typhimurium performed well in these tests, *S*. Potsdam was more resistant to biofilm formation under aerobic conditions, which could lead to adhesion to eggshells and infection of duck embryos.

Prediction of virulence genes revealed the detection of different genes associated with the pathogenicity mechanism of *Salmonella* spp. in addition to the typical genes of SPI-1 and SPI-2. *Salmonella* pathogenicity includes endotoxin, enterotoxin, and virulence effectors encoded by bacterial genetic elements. Most pathogenic *Salmonella* contains an approximately 90 kb pSLT virulence plasmid containing an 8-kb region of highly conserved five genes (*spvRABCD*) that have been reported to be associated with intracellular survival and growth (Passaris et al., 2018), *spvB, spvC* and *spvR* were found in *S*. Typhimurium (100%), and *rck* gene was identified only in *S.* Typhimurium, which encodes an outer membrane protein that enhances bacterial adhesion and invasion and confers a high level of resistance, which is consistent with the antibiotic phenotype.

In 2002, a foodborne outbreak of *Salmonella* Potsdam associated with egg salad was documented, and the organism was suspected to be related to eggs as a potential source (Unicomb et al., 2003). Since 2005, there has been an increase in the number of *S.* Potsdam isolates from different regions, with the majority obtained from waterfowl or water (Huang et al., 2014; Su et al., 2011; Tsai and Hsiang, 2005; Wang et al., 2020). Two previous cases of foodborne salmonellosis resulting in death or illness were attributed to *S*. Potsdam (Cheng et al., 2018; Cuypers et al., 2023), suggesting its potential to cause human infections and emphasizing the need for public health vigilance. Our investigations, in line with findings from goose eggs in Sichuan (Guan et al., 2022), the most common serovar observed in both datasets was *S*. Potsdam, suggesting a potential link to waterfowl, but there is still much to be explored in this serovar. The obtained knowledge may offer novel insights into their adaptive evolution under distinct geographic and environmental conditions, potentially indicating specific adaptations to waterfowl hosts, a phenomenon scarcely documented in previous studies. These observations underscore the importance of further research to unravel the complex interplay between *S*. Potsdam and its hosts, with significant implications for public health understanding and management.

This study was conducted to investigate the infection status of *Salmonella* in duck embryo eggs from Zhejiang Province, China. The findings revealed that *S*. Potsdam was the predominant serovar, indicating a previously under-recognised public health threat. The animal infection assays revealed that *S*. Potsdam exhibited a preference for ducks, which may be related to its host adaptation to the waterfowl. However, the study had some limitations: only one farm was sampled and the study was mainly conducted using a single strain. To gain a more comprehensive understanding of the sources of *Salmonella* diversity, further work is needed to expand the sample range and compare diversity, as well as to carry out more in-depth comparative genomics studies.

## Material and methods

### Sample collection and bacterial isolation

195 duck embryo eggs were obtained from a duck breeder farm (Shaoxing, Zhejiang, China) in October 2022. A decline in the health of the farm's duck flock, together with a significant increase in the number of dead embryonic eggs, prompted the farm's management to seek the help of an external researcher. Aseptic cotton swabs collected material from the egg contents and allantoic fluid. The examined samples were then enriched in sterile peptone water (BPW, Land Bridge, Beijing, China) at 37 °C for 24 hours. Subsequently, 500 μL of BPW was mixed with 5 mL of selenite cystine (SC, Land Bridge, Beijing, China) broth. After incubation at 37 °C for 24 hours, streaking was performed on xylose-lysine-desoxycholate (XLD, Land Bridge, Beijing, China) agar plates. Black colonies were selected for polymerase chain reaction (PCR) identification using the *invA* primer. Positive colonies were further confirmed through the MALDI TOF mass spectrometry system.

### Antimicrobial resistance phenotype analysis

Susceptibility to different antimicrobial agents was performed as minimum inhibitory concentration (MIC) determinations using a broth microdilution method. *Pseudomonas aeruginosa* ATCC 27853 and *Escherichia coli* ATCC 25922 were used as quality controls. This assay was performed in 96-well plates, and after incubation overnight at 37 °C, Afterwards, the obtained results were interpreted according to the criteria recommended by the Clinical and Laboratory Standards Institute guidelines (CLSL, 2021). Antimicrobial resistance phenotype analysis was completed based on previous experience (Li et al., 2022b). The Muller–Hinton broth (Land Bridge, Beijing, China) and Muller–Hinton agar (Land Bridge, Beijing, China) were used. The following 14 antimicrobials were used for susceptibility testing: gentamicin (GEN), ciprofloxacin (CIP), ampicillin (AMP), amoxicillin-clavulanic acid (AMC), Imipenem (IPM), chloramphenicol (CHL), azithromycin (AZI), tetracycline (TET), streptomycin (STR), cefotaxime (CTX), and sulfamethoxazole-trimethoprim (SXT), kanamycin (KAN), ceftriaxone (CRO), nalidixic acid (NAL).

### Whole genome sequencing and bioinformatics analysis

The procedures were completed based on previous descriptions, with minor changes (Jia et al., 2023). The bacterial genomic DNA was extracted using HiPure Bacterial DNA Kit (TIANGEN, Beijing, China). The genomic DNA of eligible quality was sequenced on the Illumina NovaSeq 6000 platform by Novogene (Beijing, China), as described previously (Elbediwi et al., 2021). The isolate genome serovars and sequence types were predicted by SISTR v1.1 (Yoshida et al., 2016) and MLST v2.19.0 (https://github.com/tseemann/mlst), respectively. Antimicrobial-resistant genes (ARGs), plasmids, and virulence genes were identified using ResFinder (ver. 3.2), PlasmidFinder (ver. 2.0), and VFDB embedded in Abricate (https://usegalaxy.org/) respectively.

The whole genome sequencing (WGS) data of 160 strains collected from EnteroBase (https://enterobase.warwick.ac.uk) was utilized for single nucleotide polymorphism (SNP) tree analysis. The SNP tree analysis and related metadata were visualized using ITOL to present the results (Li et al., 2022c). Genomic features of the investigated *Salmonella* strains were determined by running the Prokka pipeline using the Galaxy web interface. The complete genome sequences were assembled by SMRT Link v5.0.1 using nanopore sequencing data combined with Illumina sequencing data. Use proksee to annotate the genome and edit the circle map (Grant et al., 2023).

## Biofilm formation and desiccation stress assays

The biofilm formation assay was completed based on previous experience (Kang et al., 2022), with minor changes. *P. aeruginosa* ATCC 27853 was used as positive control. The biofilm formation assay was conducted using 96-well polystyrene microtiter plates. The overnight culture of each culture was adjusted to OD_620nm_ = 0.5 ± 0.02 and diluted at 1:100 in TSB broth at 37 °C for 48 hours. After incubation, the supernatant was discarded, and the unattached bacteria were washed off with ddH_2_O three times. The plates were dried at 65 °C for 20 minutes and stained with 0.4% (w/v) crystal violet for 25 minutes. Finally, the wells were washed three times using ddH_2_O. A solution of acetone and ethanol in a 1:3 (v/v) ratio was added and left to dissolve for 20 minutes. The biofilm was quantified by measuring the OD_590nm_ using a microplate reader.

For the desiccation stress assay, the culture of each strain was adjusted to an OD_620nm_ of 0.1 ± 0.02. Next, 50 μL of the suspension was added to polystyrene 96-well plates. The plates were then sealed in a container with saturated potassium acetate solution to maintain a relative humidity of 36%. After incubation at 37 °C for 24 hours, the survival rate of bacteria was calculated using the formula (CFU after 24 h treatment / initial CFU) × 100%. The experiments were conducted with three replicates per group and repeated three times.

## Animal infection assays

Specific-pathogen Free (SPF) chicken embryos were incubated at a constant temperature of 37.5 °C and humidity of 50% to 60% until they reached 16 days of age. The fresh overnight culture was adjusted to OD600nm=0.5 and then injected into the allantoic cavity at an infectious dose of 200 CFU/100μL. 100μL of PBS was administered to the embryos as a control, and untreated chicken embryos were used as a blank control, with ten embryos in each group. The embryos were observed with an egg candler every 24 hours, and the death was judged according to the integrity of the vascular system and the movement of the embryos and recorded. Finally, survival curves were plotted. The same procedure was conducted for the duck embryo infection. We used specific pathogen-free (SPF) chicks in the experiment and collected chick faeces before the experiment to confirm the absence of *Salmonella*. Each 1-day-old chick was orally infected with a dose of 10^7^ CFU in 200 μL, with an equivalent volume of oral PBS administered to the control group. Chick mortality was monitored daily for ten consecutive days, and survival curves were constructed. Ten marked chicks were used for survival observation in each group, while an additional eight chicks were sampled from each group to determine bacterial loads. On the fifth day post-infection, 3-5 chicks were randomly selected from each group for dissection, with liver, spleen, cecum, and amniotic fluid collected. Bacterial loads were determined through a 10-fold serial dilution with PBS and subsequent enumeration. A similar procedure was followed for duck infection.

## Data availability

To provide a global *S*. Potsdam context, we expanded the dataset by including 160 additional WGS genome data from the Enterobase, while others (n = 20) were preserved in our laboratory. The genomes of the *S*. Potsdam (YD86) isolates reported in this study have been deposited in the National Center for Biotechnology Information with registered BioProject number PRJNA1007938. The sequence data of the control strain (R51) is available in the NCBI database (SAMN17221882).

## Declaration of competing interest

No competing interests have been declared.

## Appendix Supplementary materials

S1 table: Summary of 180 complete *Salmonella* Potsdam genomic data used in this study, including the resistance genes and plasmids. Image, application 1

S2 table: A summary of the 40 *Salmonella* MLST, antigenic formula, antimicrobial resistance genes and plasmids is provided, along with a distribution of virulence genes and the percentage of isolates carrying virulence genes. Image, application 2
